# Circulating cell-free methylated DNA and lactate dehydrogenase release in colorectal cancer

**DOI:** 10.1186/1471-2407-14-245

**Published:** 2014-04-08

**Authors:** Alexander B Philipp, Dorothea Nagel, Petra Stieber, Rolf Lamerz, Isabel Thalhammer, Andreas Herbst, Frank T Kolligs

**Affiliations:** 1 Department of Medicine II, Ludwig-Maximilians-Universität München, Marchioninistr. 15, 81377 Munich, Germany; 2 Institute of Laboratory Medicine, Ludwig-Maximilians-Universität München, Munich, Germany

**Keywords:** Colorectal cancer, Dna methylation, Hltf, Hpp1, Neurog1, Ldh

## Abstract

**Background:**

Hypermethylation of DNA is an epigenetic alteration commonly found in colorectal cancer (CRC) and can also be detected in blood samples of cancer patients. Methylation of the genes helicase-like transcription factor (*HLTF*) and hyperplastic polyposis 1 (*HPP1*) have been proposed as prognostic, and neurogenin 1 (*NEUROG1*) as diagnostic biomarker. However the underlying mechanisms leading to the release of these genes are unclear. This study aimed at examining the possible correlation of the presence of methylated genes *NEUROG1*, *HLTF* and *HPP1* in serum with tissue breakdown as a possible mechanism using serum lactate dehydrogenase (LDH) as a surrogate marker. Additionally the prognostic impact of these markers was examined.

**Methods:**

Pretherapeutic serum samples from 259 patients from all cancer stages were analyzed. Presence of hypermethylation of the genes *HLTF*, *HPP1*, and *NEUROG1* was examined using methylation-specific quantitative PCR (MethyLight). LDH was determined using an UV kinetic test.

**Results:**

Hypermethylation of *HLTF* and *HPP1* was detected significantly more often in patients with elevated LDH levels (32% vs. 12% [p = 0.0005], and 68% vs. 11% [p < 0.0001], respectively). Also, higher LDH values correlated with a higher percentage of a fully methylated reference in a linear fashion (Spearman correlation coefficient 0.18 for *HLTF* [p = 0.004]; 0.49 [p < .0001] for *HPP1*). No correlation between methylation of *NEUROG1* and LDH was found in this study. Concerning the clinical characteristics, high levels of LDH as well as methylation of *HLTF* and *HPP1* were significantly associated with larger and more advanced stages of CRC. Accordingly, these three markers were correlated with significantly shorter survival in the overall population. Moreover, all three identified patients with a worse prognosis in the subgroup of stage IV patients.

**Conclusions:**

We were able to provide evidence that methylation of *HLTF* and especially *HPP1* detected in serum is strongly correlated with cell death in CRC using LDH as surrogate marker. Additionally, we found that prognostic information is given by both *HLTF* and *HPP1* as well as LDH. In sum, determining the methylation of *HLTF* and *HPP1* in serum might be useful in order to identify patients with more aggressive tumors.

## Background

Colorectal cancer (CRC) is the third most common cancer and the fourth most frequent cause of death from cancer worldwide with about 1.2 million cases and about 633,000 deaths in 2008 [1]. Despite significant advances in the last decades, especially patients with metastatic disease suffer from poor prognosis [2]. In addition to new therapeutic options, biomarkers are needed that allow the identification of different subgroups of patients potentially benefitting from different treatment regimens and intensity.

In many human cancers aberrant hypermethylation of CpG islands is a common epigenetic DNA modification leading to transcriptional silencing of genes that is already detectable in early stages of carcinogenesis [3]. Genes found hypermethylated in colorectal cancer have many functions, including mismatch repair, cell-cycle regulation and cell differentiation [4]. Methylated tumor DNA cannot only be found in primary colorectal cancer tissue, but can also be detected in remote media like serum or stool and potentially be used as biomarkers for various purposes [5-7]. We have previously described methylation of the genes *neurogenin 1 (NEUROG1)* in serum and *HIC1* in stool as diagnostic markers [8,9] and *helicase-like transcription factor (HLTF)* and *hyperplastic polyposis 1 (HPP1)*, also known as *transmembrane protein with EGF-like and two follistatin-like domains 2 (TMEFF2)*, as prognostic serum markers [10,11].

*NEUROG1* is a basic helix-loop-helix transcription factor which has been identified as one of the main players in neurosensory evolution and development, especially of the inner ear [12]. Moreover *NEUROG1* has been described to be frequently hypermethylated in colorectal cancers and has been proposed as a marker to classify the CpG-island methylator phenotype in colorectal cancers [13,14].

*HLTF* is a transcription factor and a member of the SWI/SNF family of chromatin-remodeling factors [15]. The physiological function of *HLTF* has not yet been fully understood, but evidence for its association with genesis and progression of cancer exists [16]. Recently *HLTF* deficiency has been reported to significantly increase the formation of small intestinal adenocarcinoma and colon cancer in mice on a *Apc*^*min/+*^mutant background and to be associated with chromosomal instability [15]. Hypermethylation of *HLTF* can commonly be found in all stages of CRC as well as in adenomas and is associated with tumor size, stage and poor prognosis [17-20]. Besides its occurrence in serum, methylated *HLTF* has also been detected in stool samples of CRC patients [21,22].

*HPP1* encodes a transmembrane protein containing epidermal growth factor and follistatin domains. While reported to function as a tumor suppressor related to the STAT1 pathway earlier [23], a recently published study failed to identify tumors in *HPP1* mutant mice [24]. Hypermethylation of *HPP1* can be detected already early in colorectal carcinogenesis [25-27]. Hyperplastic polyps and ulcerative colitis associated dysplasias as well as a several other tumor entities, including Barrett’s-associated esophageal adenocarcinoma, gastric adenocarcinoma, bladder cancer, non-small cell lung cancer and others, frequently showed *HPP1* methylation [26-32].

Lactate dehydrogenase (LDH) is essential for anaerobic glycolysis and reversably converts pyruvate to lactate. Its expression has been shown to be related to the hypoxia inducible factor HIF-1 [33-36]. Activation of the HIF pathway is a common finding in cancers [37,38]. LDH in serum is a frequently used parameter in clinical routine and is released upon cell membrane disintegration. Thus, it is an unspecific marker for tissue damage, e.g. caused by necrosis. Elevated LDH levels can be found in numerous diseases including myocardial infarction, hemolysis and malignancies [39]. Additionally LDH has been reported to be associated with more aggressive tumors and shorter survival [40-43] in CRC. In other cancer entities like testicular cancer [44,45] and aggressive non-hodgkin lymphoma [46] elevated LDH levels are used as prognostic biomarkers. Recently, LDH has been discussed as a predictive biomarker for anti-angiogenic therapies in colorectal cancer [43,47,48].

Cell death, especially necrosis, is considered to be the source of circulating cell-free DNA (cfDNA) in cancer patients [49,50]. However, the exact mechanisms leading to the release of the tumor markers discussed here with prognostic (*HLTF* and *HPP1*) or diagnostic (*NEUROG1*) information have not been examined so far. This study aimed at investigating a possible correlation of the presence of the methylated genes *NEUROG1*, *HLTF* and *HPP1* in serum with tissue breakdown as a possible release mechanism using serum lactate dehydrogenase (LDH) as a surrogate marker. Additionally, the prognostic information given by these markers was examined.

## Methods

### Patients and serum samples

Pretherapeutic serum samples from 259 patients with colorectal cancer were included in the study. For these cases clinicopathologic and follow-up data as well as pretherapeutic lactate dehydrogenase values were available. Characteristics of the cohort are shown in Table 1. All measurements were performed blinded to patient data. Blood samples were obtained pretherapeutically and underwent the following standardized preanalytical procedure: All specimens were transported by a shock absorbed tube mailing system within 15 to 30 minutes after blood drawing to the central laboratory, followed by centrifugation at 2,000 g at 4°C for 10 minutes. The supernatant serum was transferred into polypropylene cryotubes and stored frozen at −80°C. In each case, DNA methylation and lactate dehydrogenase levels were determined in the same blood sample. The study was approved by the ethical committee of the Medical Faculty of the University of Munich.

**Table 1:** Clinical features of the patient population

**Clinical feature**	**Number of patients (%)**	**Clinical feature**	**Number of patients (%)**
Total number of patients 259			
**Age**^**a**^		**Metastatic disease**	
≤ 65 years	129 (50)	M0	170 (66)
> 65 years	130 (50)	M1	89 (34)
**Sex**		**Tumor grade**^**d**^	
Male	145 (56)	G1 & G2	132 (51)
Female	114 (44)	G3 & G4	117 (45)
**Tumor size**^**b**^		**Localization**	
T1	15 (6)	Colon	122 (47)
T2	48 (19)	Sigmoid	47 (18)
T3	153 (59)	Rectum	90 (35)
T4	42 (16)	**UICC stage**	
**Nodal status**^**c**^		I	51 (20)
N0	137 (53)	II	68 (26)
N1	66 (25)	III	51 (20)
N2	50 (19)	IV	89 (34)

^a^Mean age: 64.8 years.

^b^Tumor size was unknown in 1 case.

^c^Nodal status was unknown in 6 cases.

^d^Tumor grade was unknown in 10 cases.

### DNA isolation and bisulfite conversion

The frozen serum samples were thawed at room temperature and homogenized by smoothly flipping the tube containing the serum. Genomic DNA from 200 μL of each serum sample was isolated using the High Pure Viral Nucleic Acid Kit (Roche Applied Science, Mannheim, Germany) according to the manufacturer’s instructions and eluted in 50 μl of Elution Buffer. Bisulfite conversion was performed as described previously [11].

### Analysis of DNA methylation

Bisulfite-treated DNA was analyzed by a fluorescence-based, real-time PCR assay, described previously as MethyLight [51]. Dispersed *Alu* repeats were used to control for DNA amplification and to normalize for input DNA. Primer and probe sequences are listed in Additional file 1: Table S1. PCRs were done in 20 μL volumes containing 1x PCR buffer (Qiagen, Hilden, Germany), 4 mmol/L MgCl2, 250 μmol/L deoxynucleotide triphosphate mixture, 4 μL bisulfite-treated DNA, 0.05 units/μL Taq DNA polymerase (HotStar Taq, Qiagen) along with a pair of primers and probes according to Additional file 1: Table S1. PCRs were conducted in a Mastercycler® ep realplex^4^ (Eppendorf, Hamburg, Germany) using the following conditions: 95°C for 900 s followed by 50 cycles of 95°C for 30 s, 60°C for 120 s, and 84°C for 20 s. The specificity of all reactions for methylated DNA was confirmed by separately amplifying completely methylated and unmethylated human control DNA (Chemicon, Temecula, CA) with each set of primers and probes. The percentage of fully methylated reference *(PMR)* at a specific locus was calculated as described previously [51] by dividing the gene/Alu ratio of a sample by the gene/Alu ratio of fully methylated, bisulfite-treated DNA (CpGenome™ Universal Methylated DNA, Millipore, Billerica, MA) and multiplying by 100. A gene was considered methylated if the percentage of the fully methylated reference value was > 0.

### Determination of LDH

LDH values were determined by a UV kinetic test using the Beckman Coulter AU 2700 analyser (Beckman Coulter GmbH, Krefeld, Germany) by the central laboratory of the university hospital of Munich. The upper limit of normal for this assay applied in everyday clinical routine is 250 U/l in our hospital. LDH levels above this value were defined as elevated in this study.

### Statistical analysis

All statistical analysis was done using SAS 9.3 (SAS Institute Inc., Cary, NC). Pearson’s χ^2^ test was used to explore associations between clinicopathologic features and categorized variables. Associations between categorized and continuous variables were tested by means of the Wilcoxon-Mann–Whitney test and correlations between continuous variables were examined using Spearman Correlation Coefficients. For evaluation of simultaneous influence of clinicopathologic features and methylation markers on LDH values a multivariate logistic regression model was developed. Overall survival was calculated from the date of diagnosis of the primary tumor to the date of death or end of follow-up. Univariate analysis of overall survival according to gene methylation status and LDH values was performed using the Kaplan–Meier method and log-rank tests.

## Results

### Clinicopathologic features and DNA methylation in serum

A total number of 259 serum samples were analyzed. An overview of the clinocopathologic characteristics is shown in Table 1. Methylation of *HLTF* was detected in 41 cases (16%), methylation of *HPP1* in 57 cases (22%) and methylation of *NEUROG1* in 66 cases (25%). The distribution of PMR values is demonstrated in Additional file 2: Table S2. *HLTF* methylation in the serum was significantly correlated with metastatic diseases (p = 0.013) and advanced tumor stages (p = 0.0489) as well as T4 tumors (T1-3 vs. T4, p = 0.046). A non-significant trend towards spread to lymph nodes was observed (N0 vs. N1-2, p = 0.050). *HPP1* methylation in serum was significantly correlated with larger tumor size (p < 0.001), positive nodal status (p < 0.0001), metastatic disease (p < 0.0001), tumor stage (p < .0001) as well as higher tumor grades (p = 0.0002). No significant correlation between *NEUROG1* methylation and clinicopathologic features existed. The complete distribution of the markers among the clinicopathologic features is presented in Table 2.

**Table 2:** **Distribution of LDH and methylation of *HLTF*, *HPP1 *** **and *NEUROG1 *** **among clinicopathologic features**

**Clinical feature**	**LDH ≥ 250 U/l**		**HLTF methylation**		**HPP1 methylation**		**NEUROG1 methylation**	
	**n (%)**	**P**	**n (%)**	**P**	**n (%)**	**P**	**n (%)**	**p**
**Total positive**	50 (19)		41 (16)		57 (22)		66 (25)	
**Age**^**a**^								
≤ 65 years	31 (24)		18 (14)		31 (24)		36 (28)	
> 65 years	19 (15)	0.055	23 (18)	0.410	26 (20)	0.434	30 (23)	0.372
**Sex**								
Male	26 (18)		22 (15)		34 (23)		34 (23)	
Female	24 (21)	0.528	19 (17)	0.744	23 (20)	0.528	32 (28)	0.397
**Tumor size**^**a**^								
T1	0 (0)		2 (13)		1 (7)		4 (27)	
T2	9 (19)		3 (6)		3 (6)		12 (25)	
T3	28 (18)		25 (16)		32 (21)		39 (25)	
T4	13 (31)	0.062	11 (27)	0.080	20 (48)	<.0001	11 (26)	0.999
**Nodal status**^**b**^								
N0	14 (10)		16 (12)		13 (9)		37 (27)	
N1	19 (29)		13 (20)		23 (35)		13 (20)	
N2	15 (30)	0.0006	11 (22)	0.139	18 (36)	<.0001	16 (32)	0.307
**Metastatic disease**								
M0	13 (8)		20 (12)		10 (6)		48 (28)	
M1	37 (42)	<.0001	21 (24)	0.013	47 (53)	<.0001	18 (20)	0.160
**Localization**								
Colon	25 (20)		22 (18)		33 (27)		38 (31)	
Sigmoid	9 (19)		10 (21)		8 (17)		8 (17)	
Rectum	9 (19)	0.884	9 (10)	0.151	16 (18)	0.180	20 (22)	0.114
**Tumor grade**^**c**^								
G1 & G2	22 (17)		16 (12)		16 (12)		37 (28)	
G3 & G4	25 (21)	0.344	23 (20)	0.102	37 (32)	0.0002	27 (23)	0.372
**UICC stage**								
I	6 (12)		4 (8)		2 (4)		16 (31)	
II	4 (6)		11 (16)		4 (6)		19 (28)	
III	3 (6)		5 (10)		4 (8)		13 (25)	
IV	37 (42)	<.0001	21 (24)	0.049	47 (53)	<.0001	18 (20)	0.486

^a^Tumor size was unknown in 1 case.

^b^Nodal status was unknown in 6 cases.

^c^Tumor grade was unknown in 10 cases.

LDH values ranged from 100 to 1730 U/l with a mean value of 238 U/l (standard deviation 202 U/l) and a median value of 185 U/l. A cutoff of 250 U/l, representing the upper limit of normal of the assay used, was chosen, resulting in 50 patients (19%) with elevated LDH levels. These patients suffered more frequently from T4 tumors (T1-3 vs. T4, p = 0.038), nodal and distant metastases (p = 0.0006 and p < 0.0001, respectively) as well as higher tumor stages (p < 0.0001). Additionally, a non-significant trend towards higher LDH levels in younger patients was found (p = 0.055).

### Correlation between LDH and DNA methylation in serum

First we analyzed the correlation of methylation of *HLTF, HPP1* and *NEUROG1* with LDH in a binary way. For this purpose we used a cutoff of LDH at 250 U/l as mentioned above. For the methylation markers we considered a PMR > 0 as methylation positive which has been shown previously to be reasonable for serum methylation analysis by our and other groups [10,52,53]. In the 50 samples with elevated LDH levels, methylation of *HLTF*, *HPP1,* or *NEUROG1* was detected in 16 (32%), 34 (68%), or 12 cases (24%), respectively, compared to 25 (12%), 23 (11%), or 54 (26%) in those 209 samples with normal LDH levels. Patients with elevated LDH levels revealed significantly more often methylation of *HLTF* or *HPP1* (p = 0.0005 or p < 0.0001, respectively), whereas no correlation between *NEUROG1* methylation and elevated LDH was found.

We also examined the relation of the methylation markers between each other. Methylation of *HLTF* was found significantly more often in *HPP1* positive samples (51% vs. 17%, p < 0.0001). No significant difference in the frequency of either *HLTF* or *HPP1* methylation was observed between *NEUROG1* positive and *NEUROG1* negative cases (32% vs. 24% and 26% vs. 25%, respectively).

**Figure 1:**
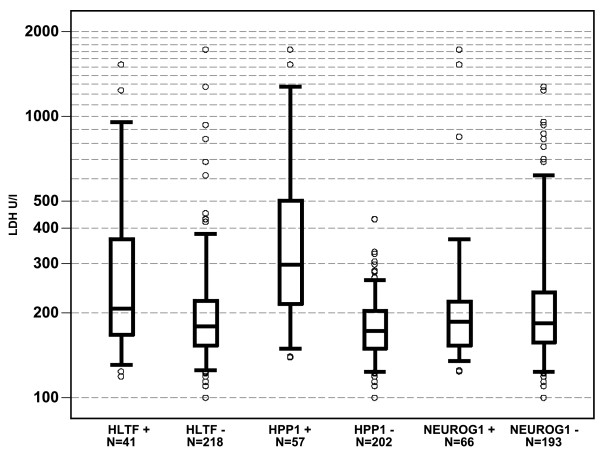
**LDH values and methylation status of *HLTF*, *HPP1 *** **and *NEUROG1 *** **(as binary variables, cutoff PMR > 0).**

In a second step, correlations were analyzed using LDH as a continous variable without cutoff. In *HPP1* positive samples significantly higher LDH levels were measured (median 298 U/l vs. 173 U/l, p < 0.0001). Patients with methylation of *HLTF* had slightly, but still significantly higher LDH levels (median 208 U/l vs. 180 U/l, p = 0.0050), while no difference was found in LDH levels between *NEUROG1* positive and negative samples (median 187 U/L vs. 184 U/l, p = 0.95). Figure 1 provides a more detailed view on the distribution of LDH levels among the three methylation markers.

Additionally, we tested *HLTF*, *HPP1* and *NEUROG1* as continuous variables without cutoff using the PMR values and calculated univariate Spearman correlation coefficients. As in the analyses before, *HLTF* and *HPP1* showed significant correlation with LDH, while *NEUROG1* did not. All linear correlation coefficients and p-values are presented in Table 3.

**Table 3:** **Linear Spearman correlation coefficients for the percentage of fully methylated reference (PMR) of *HLTF*, *HPP1 *** **and *NEUROG1, *** **and LDH levels among each other**

	**PMR HLTF**	**PMR HPP1**	**PMR NEUROG1**	**LDH**
**PMR HLTF**	1.0	-	-	-
**PMR HPP1**	0.32 (p < .0001)	1.0	-	-
**PMR NEUROG1**	0.05 (p = 0.41)	−0.00 (p = 0.97)	1.0	-
**LDH**	0.18 (p = 0.004)	0.49 (p < .0001)	0.01 (p = 0.85)	1.0

### Multivariate model

Next, a multivariate model was developed using logistic regression analysis with LDH values higher than 250 U/l as target variable. *HPP1* and *HLTF* methylation as binary parameters, i.e. with a PMR > 0, as well as clinicopathological features were entered as independent variables. Only presence of distant metastases and *HPP1* correlated significantly and independently with elevated LDH levels higher than 250 U/l. The odds ratios were 3.1 for metastatic disease (95% CI 1.3-7.2, p = 0.009) and 9.5 for *HPP1* methylation (95% CI 4.2-21.9, p < 0.0001).

### Survival analysis

We earlier reported methylation of *HLTF* and *HPP1* to be independent prognostic markers in metastastatic colorectal cancer [11]. On the other hand, elevated LDH levels have been described to be linked to shorter survival [54]. Thus we compared methylation of *HLTF* and *HPP1* with LDH as prognostic factors in our patient population.

**Figure 2:**
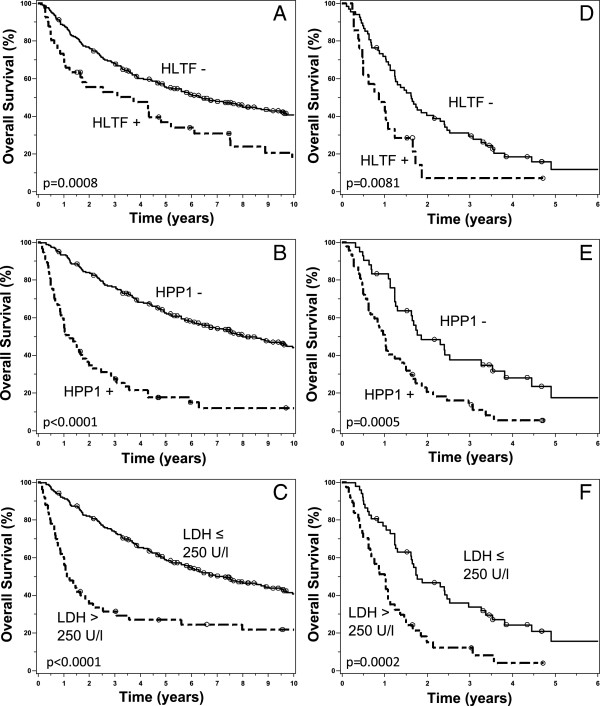
**Kaplan-Meier plots of overall survival. A-C**: Overall survival for all patients according to methylation status of *HLTF***(A)**, *HPP1***(B)** and high LDH levels > 250 U/l **(C)**, respectively. **D**-**F**: Overall survival for stage IV patients patients according to methylation status of *HLTF***(D)**, *HPP1***(E)** and high LDH levels > 250 U/l **(F)**, respectively.

As reported earlier [11] methylation of *HLTF* and *HPP1* was associated with a higher mortality. In the current study, the median survival was 6.4 years (95% CI 4.9-9.0) and 8.0 years (95% CI 6.1-11.2) for *HLTF*- and *HPP1-*negative cases compared to 3.7 years (95% CI 1.1-5.2) and 1.2 years (95% CI 0.9-1.9) in case of positivity for *HLTF* or *HPP1* methylation (p = 0.0008 and p < 0.0001), respectively (Figure 2A, 2B). LDH levels above a cutoff of 250 U/l were associated with shorter overall survival (median survival 1.1 years, 95% CI 0.9-2.0) compared to low LDH levels (median survival 7.2 years, 95% CI 5.6-9.6) (p < 0.0001) (Figure 2C).

Next, we evaluated the prognostic significance stratified by tumor stage. For patients with UICC stage I-III no significant difference in overall survival, neither for LDH (p = 0.41) nor for *HLTF* and *HPP1* (p = 0.41 and p = 0.08, respectively), was found. However, in stage IV *HLTF* methylation positive patients showed a median survival of 0.86 years (95% CI 0.5-1.2) versus 1.6 years (95% CI 1.2-2.3) for *HLTF* negative cases (p = 0.0081; Figure 2D). For *HPP1* positive and negative cases the median survival was 1.0 years (95% CI 0.6-1.4) and 1.8 years (95% CI 1.2-3.3), respectively (p = 0.0005; Figure 2E). For LDH, elevated levels > 250 U/l were associated with shorter median survival (1.0 years, 95% CI 0.6-1.2, vs. 1.8 years, 95% CI 1.3-2.5; p = 0.0002; Figure 2F).

## Discussion

In this study we examined the correlation between cell damage using LDH as a surrogate marker and the methylation status of three genes which have previously been proposed as prognostic (*HLTF, HPP1*) [10,11] or diagnostic (*NEUROG1*) [8] biomarkers for patients with CRC.

Our data confirm our previous findings that methylation of *HLTF* or *HPP1* in serum is found more often in patients with advanced stages of colorectal cancer, especially in those with distant metastases, whereas no correlation between methylation of *NEUROG1* and any clinicopathologic data was found. While methylation of *HLTF* was only correlated with metastastatic disease, methylation of *HPP1* was also associated with local tumor extent and nodal status as well as tumor grade with high statistic significance.

It is well known that patients with elevated serum levels of LDH tend to have more aggressive tumors and a shorter survival time [40-43]. Consistent with the literature high LDH levels in our data were significantly correlated with advanced tumor stages as well as nodal and distant metastases. Trends towards larger tumor size and younger age were observed but did not reach statistical significance.

Cell death associated mechanisms like apoptosis or, especially in cancer, necrosis have been suggested as main sources for cell-free DNA (cfDNA) in the blood, but other mechanisms like physiological active release have been described as well (for reviews see refs. [49,50]). In this study we found methylation of *HLTF* and, even to a higher degree, *HPP1* to be correlated with elevated LDH levels. This finding was robust, as it was confirmed by different statistical methods. Given that elevated LDH indicates cell membrane damage, this observation might be a hint that methylated *HLTF* and *HPP1* DNA is released by tumor cells undergoing cell death. The fact that necrosis tends to be found more often in larger, more aggressive tumours and advanced cancer stages [55,56], which was likewise the case for LDH as well as methylated *HLTF* and *HPP1* in our data, also suggests an interrelation.

For *NEUROG1*, on the other hand, hypermethylation in serum was detectable independently of LDH levels and tumor stage. This is consistent with earlier analyses revealing methylation of *NEUROG1* in primary tissue not to be associated with tumor stage (A.P. and F.K., data not published). Hence the observed correlation between DNA methylation in serum and LDH seems not to be linked to global methylation levels and cell death alone. Besides the methylation status of distinct genes, other parameters influencing this observation might include DNA integrity and stability of the respective segments as well as still unknown factors. Therefore it seems likely that tumor cell death might not be the only mechanism by which methylated tumor DNA is released to the blood.

In addition to the correlation analysis we examined the prognostic significance of the methylation markers *HPP1* and *HLTF* as well as of LDH. All three markers were significantly associated with worse overall survival. This could be attributed to the fact that all three markers are found more frequently in advanced cancer stages. However, earlier analyses [11] as well as the survival data presented here furthermore divide patients with already metastasized disease into two subgroups with better or worse prognosis, respectively.

## Conclusion

In conclusion we were able to provide evidence that methylation of *HLTF* and especially *HPP1* detected in serum is strongly correlated with cell death in colorectal cancer using LDH as surrogate marker. However, this finding was specific for those two genes and did not occur for *NEUROG1*, suggesting that mechanisms other than release by membrane disintegration could be responsible for the occurrence of cell-free DNA in blood of CRC patients. Additionally, we found that prognostic information is given by both *HLTF* and *HPP1* as well as LDH. In sum, determining the methylation of *HLTF* and *HPP1* in serum might be useful in order to identify patients with more aggressive tumors. Future research needs to further clarify the underlying biological mechanisms and to validate methylated cell-free circulating DNA as a biomarker for colorectal cancer.

## Abbreviations

cfDNA: Cell-free deoxyribonucleic acid; CI: Confidence interval; CIMP: CpG island methylator phenotype; CRC: Colorectal cancer; HIF: Hypoxia inducible factor; HLTF: Helicase-like transcription factor; HPP1: Hyperplastic polyposis; LDH: Lactate dehydrogenase; NEUROG1: Neurogenin 1; PCR: Polymerase chain reaction; PMR: Percentage of fully methylated reference; UICC: Union for international cancer control; UV: Ultraviolet.

## Competing interests

The authors declare that they have no competing interest.

## Authors’ contributions

Sample collection and experiments: AP, IT, PS, and RL; data analysis and interpretation: AP, DN, PS, and FK; study design and preparation of the manuscript: AP, AH, and FK. All authors read and approved the final manuscript.

## Pre-publication history

The pre-publication history for this paper can be accessed here:

http://www.biomedcentral.com/1471-2407/14/245/prepub

## Supplementary Material

Additional file 1 MethyLight Reaction Details. Click here for file

Additional file 2 **Distribution of the percentage of fully methylated reference (PMR) of *HLTF, HPP1 *** **and *NEUROG1.***
Click here for file

## References

[B1] Ferlay J Shin HR Bray F Forman D Mathers C Parkin DM Estimates of worldwide burden of cancer in 2008: GLOBOCAN 2008 Int J Cancer 2010 127 2893 2917 10.1002/ijc.25516 21351269

[B2] O’Connell JB Maggard MA Ko CY Colon cancer survival rates with the new American Joint Committee on Cancer sixth edition staging J Natl Cancer Inst 2004 96 1420 1425 10.1093/jnci/djh275 15467030

[B3] Jones PA Baylin SB The epigenomics of cancer Cell 2007 128 683 692 10.1016/j.cell.2007.01.029 17320506 PMC3894624

[B4] Baylin SB Ohm JE Epigenetic gene silencing in cancer - a mechanism for early oncogenic pathway addiction? Nat Rev Cancer 2006 6 107 116 10.1038/nrc1799 16491070

[B5] Duffy MJ Napieralski R Martens JWM Span PN Spyratos F Sweep FCGJ Brunner N Foekens JA Schmitt M Methylated genes as new cancer biomarkers Eur J Cancer 2009 45 335 346 10.1016/j.ejca.2008.12.008 19138839

[B6] Kim MS Lee J Sidransky D DNA methylation markers in colorectal cancer Cancer Metastasis Rev 2010 29 181 206 10.1007/s10555-010-9207-6 20135198

[B7] Herbst A Kolligs FT Detection of DNA hypermethylation in remote media of patients with colorectal cancer: new biomarkers for colorectal carcinoma Tumour Biol 2012 33 297 305 10.1007/s13277-012-0346-y 22362383

[B8] Herbst A Rahmig K Stieber P Philipp A Jung A Ofner A Crispin A Neumann J Lamerz R Kolligs FT Methylation of NEUROG1 in Serum Is a Sensitive Marker for the Detection of Early Colorectal Cancer Am J Gastroenterol 2011 106 1110 1118 10.1038/ajg.2011.6 21326223

[B9] Lenhard K Bommer GT Asutay S Schauer R Brabletz T Göke B Lamerz R Kolligs FT Analysis of promoter methylation in stool: a novel method for the detection of colorectal cancer Clin Gastroenterol Hepatol 2005 3 142 149 10.1016/S1542-3565(04)00624-X 15704048

[B10] Wallner M Herbst A Behrens A Crispin A Stieber P Göke B Lamerz R Kolligs FT Methylation of serum DNA is an independent prognostic marker in colorectal cancer Clin Cancer Res 2006 12 7347 7352 10.1158/1078-0432.CCR-06-1264 17189406

[B11] Philipp AB Stieber P Nagel D Neumann J Spelsberg F Jung A Lamerz R Herbst A Kolligs FT Prognostic role of methylated free circulating DNA in colorectal cancer Int J Cancer 2012 131 2308 2319 10.1002/ijc.27505 22362391

[B12] Pan N Kopecky B Jahan I Fritzsch B Understanding the evolution and development of neurosensory transcription factors of the ear to enhance therapeutic translation Cell Tissue Res 2012 349 415 432 10.1007/s00441-012-1454-0 22688958 PMC3508675

[B13] Weisenberger DJ Siegmund KD Campan M Young J Long TI Faasse MA Kang GH Widschwendter M Weener D Buchanan D Koh H Simms L Barker M Leggett B Levine J Kim M French AJ Thibodeau SN Jass J Haile R Laird PW CpG island methylator phenotype underlies sporadic microsatellite instability and is tightly associated with BRAF mutation in colorectal cancer Nat Genet 2006 38 787 793 10.1038/ng1834 16804544

[B14] Ogino S Cantor M Kawasaki T Brahmandam M Kirkner GJ Weisenberger DJ Campan M Laird PW Loda M Fuchs CS CpG island methylator phenotype (CIMP) of colorectal cancer is best characterised by quantitative DNA methylation analysis and prospective cohort studies Gut 2006 55 1000 1006 10.1136/gut.2005.082933 16407376 PMC1856352

[B15] Sandhu S Wu X Nabi Z Rastegar M Kung S Mai S Ding H Loss of HLTF function promotes intestinal carcinogenesis Mol Cancer 2012 11 18 10.1186/1476-4598-11-18 22452792 PMC3337324

[B16] Debauve G Capouillez A Belayew A Saussez S The helicase-like transcription factor and its implication in cancer progression Cell Mol Life Sci 2008 65 591 604 10.1007/s00018-007-7392-4 18034322 PMC11131614

[B17] Moinova HR Chen W-D Shen L Smiraglia D Olechnowicz J Ravi L Kasturi L Myeroff L Plass C Parsons R Minna J Willson JKV Green SB Issa J-P Markowitz SD HLTF gene silencing in human colon cancer Proc Natl Acad Sci U S A 2002 99 4562 4567 10.1073/pnas.062459899 11904375 PMC123687

[B18] Bai AHC Tong JHM To K-F Chan MWY Man EPS Lo K-W Lee JFY Sung JJY Leung WK Promoter hypermethylation of tumor-related genes in the progression of colorectal neoplasia Int J Cancer 2004 112 846 853 10.1002/ijc.20485 15386372

[B19] Hibi K Nakao A Highly-methylated colorectal cancers show poorly-differentiated phenotype Anticancer Res 2006 26 4263 4266 17201142

[B20] Kim Y Petko Z Dzieciatkowski S Lin L Ghiassi M Stain S Chapman WC Washington MK Willis J Markowitz SD Grady WM CpG island methylation of genes accumulates during the adenoma progression step of the multistep pathogenesis of colorectal cancer Genes Chromosomes Cancer 2006 45 781 789 10.1002/gcc.20341 16708352

[B21] Leung WK To K-F Man EPS Chan MWY Bai AHC Hui AJ Chan FKL Lee JFY Sung JJY Detection of epigenetic changes in fecal DNA as a molecular screening test for colorectal cancer: a feasibility study Clin Chem 2004 50 2179 2182 10.1373/clinchem.2004.039305 15502094

[B22] Leung WK To K-F Man EPS Chan MWY Hui AJ Ng SSM Lau JYW Sung JJY Detection of hypermethylated DNA or cyclooxygenase-2 messenger RNA in fecal samples of patients with colorectal cancer or polyps Am J Gastroenterol 2007 102 1070 1076 10.1111/j.1572-0241.2007.01108.x 17378912

[B23] Elahi A Zhang L Yeatman TJ Gery S Sebti S Shibata D HPP1-mediated tumor suppression requires activation of STAT1 pathways Int J Cancer 2008 122 1567 1572 18059030 10.1002/ijc.23202

[B24] Chen TR Wang P Carroll LK Zhang Y Han B-X Wang F Generation and characterization of Tmeff2 mutant mice Biochem Biophys Res Commun 2012 425 189 194 10.1016/j.bbrc.2012.07.064 22828515 PMC3428475

[B25] Ebert MP Mooney SH Tonnes-Priddy L Lograsso J Hoffmann J Chen J Röcken C Schulz H-U Malfertheiner P Lofton-Day C Hypermethylation of the TPEF/HPP1 Gene in Primary and Metastatic Colorectal Cancers Neoplasia 2005 7 771 778 10.1593/neo.05235 16207479 PMC1501884

[B26] Young J Biden KG Simms LA Huggard P Karamatic R Eyre HJ Sutherland GR Herath N Barker M Anderson GJ Fitzpatrick DR Ramm GA Jass JR Leggett BA HPP1: a transmembrane protein-encoding gene commonly methylated in colorectal polyps and cancers Proc Natl Acad Sci U S A 2001 98 265 270 10.1073/pnas.98.1.265 11120884 PMC14579

[B27] Sato F Shibata D Harpaz N Xu Y Yin J Mori Y Wang S Olaru A Deacu E Selaru FM Kimos MC Hytiroglou P Young J Leggett B Gazdar AF Toyooka S Abraham JM Meltzer SJ Aberrant methylation of the HPP1 gene in ulcerative colitis-associated colorectal carcinoma Cancer Res 2002 62 6820 6822 12460892

[B28] Saito S Kato J Hiraoka S Horii J Suzuki H Higashi R Kaji E Kondo Y Yamamoto K DNA methylation of colon mucosa in ulcerative colitis patients: Correlation with inflammatory status Inflamm Bowel Dis 2011 17 1955 1965 10.1002/ibd.21573 21830274

[B29] Eads CA Lord RV Kurumboor SK Wickramasinghe K Skinner ML Long TI Peters JH DeMeester TR Danenberg KD Danenberg PV Laird PW Skinner KA Fields of aberrant CpG island hypermethylation in Barrett’s esophagus and associated adenocarcinoma Cancer Res 2000 60 5021 5026 11016622

[B30] Ivanauskas A Hoffmann J Jonaitis LV Markelis R Juozaityte E Kupcinskas L Lofton-Day C Röcken C Malfertheiner P Distinct TPEF/HPP1 gene methylation patterns in gastric cancer indicate a field effect in gastric carcinogenesis Dig Liver Dis 2008 40 920 926 10.1016/j.dld.2008.05.004 18799374

[B31] Hellwinkel OJC Kedia M Isbarn H Budäus L Friedrich MG Methylation of the TPEF- and PAX6-promoters is increased in early bladder cancer and in normal mucosa adjacent to pTa tumours BJU Int 2008 101 753 757 10.1111/j.1464-410X.2007.07322.x 18070176

[B32] Lee SM Park JY Kim DS Methylation of TMEFF2 gene in tissue and serum DNA from patients with non-small cell lung cancer Mol Cells 2012 34 171 176 10.1007/s10059-012-0083-5 22814847 PMC3887809

[B33] Semenza GL Roth PH Fang HM Wang GL Transcriptional regulation of genes encoding glycolytic enzymes by hypoxia-inducible factor 1 J Biol Chem 1994 269 23757 23763 8089148

[B34] Firth JD Ebert BL Pugh CW Ratcliffe PJ Oxygen-regulated control elements in the phosphoglycerate kinase 1 and lactate dehydrogenase A genes: similarities with the erythropoietin 3’ enhancer Proc Natl Acad Sci U S A 1994 91 6496 6500 10.1073/pnas.91.14.6496 8022811 PMC44229

[B35] Firth JD Ebert BL Ratcliffe PJ Hypoxic regulation of lactate dehydrogenase A. Interaction between hypoxia-inducible factor 1 and cAMP response elements J Biol Chem 1995 270 21021 21027 10.1074/jbc.270.36.21021 7673128

[B36] Weidemann A Johnson RS Biology of HIF-1alpha Cell Death Differ 2008 15 621 627 10.1038/cdd.2008.12 18259201

[B37] Maxwell PH Pugh CW Ratcliffe PJ Activation of the HIF pathway in cancer Curr Opin Genet Dev 2001 11 293 299 10.1016/S0959-437X(00)00193-3 11377966

[B38] Keith B Johnson RS Simon MC HIF1α and HIF2α: sibling rivalry in hypoxic tumour growth and progression Nat Rev Cancer 2012 12 9 22 22169972 10.1038/nrc3183 PMC3401912

[B39] Huijgen HJ Sanders GT Koster RW Vreeken J Bossuyt PM The clinical value of lactate dehydrogenase in serum: a quantitative review Eur J Clin Chem Clin Biochem 1997 35 569 579 9298346

[B40] Mekenkamp LJM Koopman M Teerenstra S van Krieken JHJM Mol L Nagtegaal ID Punt CJA Clinicopathological features and outcome in advanced colorectal cancer patients with synchronous vs metachronous metastases Br J Cancer 2010 103 159 164 10.1038/sj.bjc.6605737 20551951 PMC2906733

[B41] De Gramont A Figer A Seymour M Homerin M Hmissi A Cassidy J Boni C Cortes-Funes H Cervantes A Freyer G Papamichael D Le Bail N Louvet C Hendler D de Braud F Wilson C Morvan F Bonetti A Leucovorin and fluorouracil with or without oxaliplatin as first-line treatment in advanced colorectal cancer J Clin Oncol 2000 18 2938 2947 10944126 10.1200/JCO.2000.18.16.2938

[B42] Wu X Ma F Wang X-L Serological diagnostic factors for liver metastasis in patients with colorectal cancer World J Gastroenterol 2010 16 4084 4088 10.3748/wjg.v16.i32.4084 20731024 PMC2928464

[B43] Scartozzi M Giampieri R Maccaroni E Del Prete M Faloppi L Bianconi M Galizia E Loretelli C Belvederesi L Bittoni A Cascinu S Pre-treatment lactate dehydrogenase levels as predictor of efficacy of first-line bevacizumab-based therapy in metastatic colorectal cancer patients Br J Cancer 2012 106 799 804 10.1038/bjc.2012.17 22315053 PMC3305976

[B44] International Germ Cell Cancer Collaborative Group Germ Cell Consensus Classification: a prognostic factor-based staging system for metastatic germ cell cancers. International Germ Cell Cancer Collaborative Group J Clin Oncol 1997 15 594 603 9053482 10.1200/JCO.1997.15.2.594

[B45] Krege S Beyer J Souchon R Albers P Albrecht W Algaba F Bamberg M Bodrogi I Bokemeyer C Cavallin-Ståhl E Classen J Clemm C Cohn-Cedermark G Culine S Daugaard G De Mulder PHM De Santis M de Wit M de Wit R Derigs HG Dieckmann K Dieing A Droz J Fenner M Fizazi K Flechon A Fosså SD del Muro XG Gauler T Geczi L European consensus conference on diagnosis and treatment of germ cell cancer: a report of the second meeting of the European Germ Cell Cancer Consensus group (EGCCCG): part I Eur Urol 2008 53 478 496 10.1016/j.eururo.2007.12.024 18191324

[B46] A predictive model for aggressive non-Hodgkin’s lymphoma. The International Non-Hodgkin's Lymphoma Prognostic Factors Project N Engl J Med 1993 329 987 994 8141877 10.1056/NEJM199309303291402

[B47] Hecht JR Trarbach T Hainsworth JD Major P Jäger E Wolff RA Lloyd-Salvant K Bodoky G Pendergrass K Berg W Chen B-L Jalava T Meinhardt G Laurent D Lebwohl D Kerr D Randomized, placebo-controlled, phase III study of first-line oxaliplatin-based chemotherapy plus PTK787/ZK 222584, an oral vascular endothelial growth factor receptor inhibitor, in patients with metastatic colorectal adenocarcinoma J Clin Oncol 2011 29 1997 2003 10.1200/JCO.2010.29.4496 21464406

[B48] Van Cutsem E Bajetta E Valle J Köhne C-H Randolph Hecht J Moore M Germond C Berg W Chen B-L Jalava T Lebwohl D Meinhardt G Laurent D Lin E Randomized, placebo-controlled, phase III study of oxaliplatin, fluorouracil, and leucovorin with or without PTK787/ZK 222584 in patients with previously treated metastatic colorectal adenocarcinoma J Clin Oncol 2011 29 2004 2010 10.1200/JCO.2010.29.5436 21464401

[B49] Jung K Fleischhacker M Rabien A Cell-free DNA in the blood as a solid tumor biomarker–a critical appraisal of the literature Clin Chim Acta 2010 411 1611 1624 10.1016/j.cca.2010.07.032 20688053

[B50] Schwarzenbach H Hoon DSB Pantel K Cell-free nucleic acids as biomarkers in cancer patients Nat Rev Cancer 2011 11 426 437 10.1038/nrc3066 21562580

[B51] Eads CA Danenberg KD Kawakami K Saltz LB Blake C Shibata D Danenberg PV Laird PW MethyLight: a high-throughput assay to measure DNA methylation Nucleic Acids Res 2000 28 E32 10.1093/nar/28.8.e32 10734209 PMC102836

[B52] Müller HM Widschwendter A Fiegl H Ivarsson L Goebel G Perkmann E Marth C Widschwendter M DNA methylation in serum of breast cancer patients: an independent prognostic marker Cancer Res 2003 63 7641 7645 14633683

[B53] Ebert MP Model F Mooney S Hale K Lograsso J Tonnes-Priddy L Hoffmann J Csepregi A Röcken C Molnar B Schulz H-U Malfertheiner P Lofton-Day C Aristaless-like homeobox-4 gene methylation is a potential marker for colorectal adenocarcinomas Gastroenterology 2006 131 1418 1430 10.1053/j.gastro.2006.08.034 17101318

[B54] Tas F Aykan F Alici S Kaytan E Aydiner A Topuz E Prognostic factors in pancreatic carcinoma: serum LDH levels predict survival in metastatic disease Am J Clin Oncol 2001 24 547 550 10.1097/00000421-200112000-00003 11801751

[B55] Pollheimer MJ Kornprat P Lindtner RA Harbaum L Schlemmer A Rehak P Langner C Tumor necrosis is a new promising prognostic factor in colorectal cancer Hum Pathol 2010 41 1749 1757 10.1016/j.humpath.2010.04.018 20869096

[B56] Richards CH Roxburgh CSD Anderson JH McKee RF Foulis AK Horgan PG McMillan DC Prognostic value of tumour necrosis and host inflammatory responses in colorectal cancer Br J Surg 2012 99 287 294 10.1002/bjs.7755 22086662

