# Supplementary material for: Circulating cell-free methylated DNA and lactate dehydrogenase release in colorectal cancer
Source: BMC Cancer. 2014 Apr 8;14:245. doi: 10.1186/1471-2407-14-245 (PMC4021086; doi:10.1186/1471-2407-14-245)
Supplement: Additional file 1 — MethyLight Reaction Details. [file 1471-2407-14-245-S1.doc]

Additional file 1: Table S1

MethyLight Reaction Details

| **Gene ID** | **Amplicon Size [bp]** | **Forward Primer** | | **Reverse Primer** | | **Probe Oligo** | |
| --- | --- | --- | --- | --- | --- | --- | --- |
|  |  | **Sequence (5′-3′)** | **Concen­tration [nmol/l]** | **Sequence (5′-3′)** | **Concen­tration [nmol/l]** | **Sequence (5′ 6-FAM / 3′ BHQ-1M´; 5′-3′)** | **Concen­tration [nmol/l]** |
| Alu | 98 | GGTTAGGTATAGTGGTTTATATTTGTAATTTTAGTA | 600 | ATTAACTAAACTAATCTTAAACTCCTAACCTCA | 600 | CCTACCTTAACCTCCC | 300 |
| HLTF | 95 | CGGCGTTCGGAATTTGTT | 455 | AAACGCCTCGACTCCCCTAA | 455 | AGGAGGCGTATCGAGGCGGTTCG | 180 |
| HPP1 | 87 | GTTATCGTCGTCGTTTTTGTTGTC | 455 | GACTTCCGAAAAACACAAAATCG | 455 | CCGAACAACGGACTACTAAACATCCCGCG | 250 |
| NEUROG1 | 89 | CGTGTAGCGTTCGGGTATTTGTA | 600 | CGATAATTACGAACACACTCCGAAT | 600 | CGATAACGACCTCCCGCGAACATAAA | 300 |
