# Supplementary material for: Circulating cell-free methylated DNA and lactate dehydrogenase release in colorectal cancer
Source: BMC Cancer. 2014 Apr 8;14:245. doi: 10.1186/1471-2407-14-245 (PMC4021086; doi:10.1186/1471-2407-14-245)
Supplement: Additional file 2 — Distribution of the percentage of fully methylated reference (PMR) of HLTF, HPP1 and NEUROG1. [file 1471-2407-14-245-S2.doc]

*Additional file 2: Table S2
Distribution of the percentage of fully methylated reference (PMR) of HLTF, HPP1 and NEUROG1*

| **PMR** | **HLTF** | **HPP1** | **NEUROG1** |
| --- | --- | --- | --- |
| 0 | 218 (84%) | 202 (78%) | 193 (75%) |
| > 0 - < 0.01 | 23 (9%) | 21 (8%) | 14 (5%) |
| ≥ 0.01 - < 0.1 | 9 (3%) | 24 (9%) | 32 (12%) |
| ≥ 0.1 | 9 (3%) | 12 (5%) | 20 (8%) |
